# Stage 1 development of a patient-reported experience measure (PREM) for chronic obstructive pulmonary disease (COPD)

**DOI:** 10.1038/s41533-017-0047-5

**Published:** 2017-07-24

**Authors:** Susan Walker, Sharon Andrew, Matthew Hodson, C. Michael Roberts

**Affiliations:** 1 Faculty of Health, Social Care and Education, Anglia Ruskin University, Chelmsford, CMI 1SQ UK; 2 Department of Nursing & Midwifery, College of Health and Biomedicine, Victoria University, MEL 8001, Australia; 3 Honorary Respiratory Nurse Consultant, Homerton University Hospital, London, E9 6SR UK; 4 Education and Workforce and Comorbidities Programme, UCL partners, 3rd Floor 170 Tottenham Court Road, London, W1T 7HA UK

## Abstract

The study aimed to explore patients’ experience of living with chronic obstructive pulmonary disease and their perspective of their community healthcare for chronic obstructive pulmonary disease to extract affective responses in order to develop potential items for a patient-reported experience measure for chronic obstructive pulmonary disease. Qualitative face-face interviews were conducted, in the community, with 64 patients with chronic obstructive pulmonary disease recruited from General Practices and Breathe-Easy community groups in the Outer North East, East and City areas of London and Essex, UK. A two phase analysis of the qualitative data was conducted to identify themes arising from patients’ description of living with chronic obstructive pulmonary disease and their perceptions of their community healthcare and subsequently the affective responses underlying the themes raised by patients, which gave emotional colour to the themes, bringing the thematic analysis closer to the subjective patient experience. Five themes were identified from the interview data: ‘Journey to diagnosis’; ‘Smoking’; ‘Usual care’; ‘My everyday life’; and ‘Exacerbations’. Twenty-one affective responses were identified and categorised as either ‘negative’, ‘positive’ or ‘bivalent’. ‘Frustration’, a negative affective response was prevalent in four themes. ‘Gratitude’, ‘hope’ and ‘happiness/enjoyment’ were among the more positive responses more prevalent across several themes. By conducting a novel two-way analysis (thematic and affective) it was possible to identify themes and affective responses that were aligned to those themes. This enabled the development of 38 chronic obstructive pulmonary disease-specific experience items to take forward for further testing including item reduction and validity and reliability in the next stage of the patient-reported experience measure development.

## Chronic lung disease: Giving importance to patients’ emotions

An exploration of patient perceptions of living with chronic lung disease will help develop a new patient reported experience scale. Healthcare services are aiming to provide effective patient-centered care for those with chronic obstructive pulmonary disease (COPD). Such care strategies require structured, validated patient feedback scales to facilitate accurate communication between patients, carers and healthcare professionals. Susan Walker at Anglia Ruskin University in Chelmsford, UK, and co-workers analyzed qualitative data from interviews with 64 COPD patients in London and Essex regarding their emotions and perceptions of living with COPD, with the aim of creating a patient reported experience measure, or PREM. Initial results identified five themes—including ‘journey to diagnosis’ and ‘everyday life’—and twenty-one affective responses, ranging from negative to positive. The team will take these results forward for further validation.

## Introduction

The involvement of patients, the public including service users, and carers is increasingly sought with the expansion of the patient-centric approach to healthcare. Involvement ranges from giving feedback about healthcare received from an organisation to informing aspects of health research. Feedback is often sought through the use of structured questionnaires with tick-box scales.

Many scales have been developed for use with patients and one area of particular interest has been the measurement of patient satisfaction with healthcare received. There is a recognition, however, that the information in patient satisfaction scales while important for an organisation, for quality assurance and patient safety, may not be seen as important by patients whose priorities may differ from those of a healthcare organisation.^1, 2^

There has been a slow evolution of scales from generic patient satisfaction to condition-specific patient experience scales. In tandem with this change has been the collection of the patient perspective on outcomes from treatment received called patient-reported outcome measures (PROMS). In the UK, PROMS have been used in National Service Hospitals (NHS) for various elective surgical procedures including knee and hip replacement, hernia repair and varicose vein treatments, with patients completing the PROMS before and after the procedure.^1^ International researchers have developed other condition-specific PROMS for example hypertension^3^ and liver cirrhosis.^4^ PROMS are used to identify the effectiveness of a medical care or procedure from the patient perspective.^1, 4^

While there has been considerable research on designing and deploying PROMS notably within specific surgical procedures there has been very little research or practical application of patient-reported experience measures (PREMs).^1^

Reviews indicate that condition-specific PROMS for chronic obstructive pulmonary disease (COPD) are better at discriminating between different levels of COPD severity than generic scales,^5, 6^ and were rated as have better evidence synthesis for psychometric properties compared with generic scales.^7^ There are a number of COPD-specific PROMS that are completed by patients and collect evidence about overall respiratory status such as the COPD Assessment Test (CAT™),^8^ or about response to COPD symptoms such as the breathlessness, cough and sputum scale.^9^

In the UK the Department of Health has produced a national service strategy document, supported by NICE quality standards, which suggests measuring both patients outcomes and experiences^10^

In terms of PREMS, while generic measures of patient experience exist and are important, they risk losing elements of a patient’s experience that are specific or weighted towards a particular disease (e.g., COPD) that is the dominant reason for a patient to seek healthcare assistance. This suggests that patient-reported experience measures (PREM) are needed in addition to generic experience scales.

Since there are currently no COPD-specific scales or instruments, which measure patient experience this requires the development of such a scale.^11^ This paper reports efforts to develop the underlying themes of what a PREM tool would reflect of the experience of community long-term care for COPD.

In this context ‘experience’ is taken to refer to a wider concept than linearly measured satisfaction or narrowly focussed outcomes, to attempt to learn about how the patient subjectively feel about their condition and whole of the care episode in a holistic manner including ‘affective’ or emotional responses.

The affective experience of a condition and/or its treatment can differ from the outcomes measured on a PROM. For instance a patient may be satisfied with the outcome of a procedure but have found the experience frightening. Similarly, a patient may feel understood when seeking help for an exacerbation for COPD, even though the outcome was an admission to hospital (i.e., a ‘poor’ outcome). The response to healthcare received can evoke strong emotions in patients. While the importance of the affective domain is recognised in various aspects of health such as illness perceptions,^12^ it is not a domain that is generally used in the measurement of health satisfaction questionnaires. In a move away from this traditional approach in the medical literature where emotions or affective domain are not generally measured, our aim was to explore patient perceptions about their health and healthcare received with a focus on capturing what was important to patients in their care while preserving emotions underlying the language used by those patients. We considered that this would add an additional dimension to our understanding of patients’ experiences around COPD, living with the disease and their interactions with the healthcare system and healthcare personnel. The concept ‘*affect*’ has been used broadly to include emotions and feelings expressed by patients.

In 2011, the North East London, North Central London and Essex (NECLES) Health Innovation and Education Cluster (HIEC) in collaboration with The Royal College of Physicians, British Thoracic Society, British Lung Foundation, Picker Institute, City University and Anglia Ruskin University undertook preliminary research to inform the development of a PREM for use in all people with COPD.^13^

This paper describes the preliminary Stage 1 development of a PREM for COPD, which developed the key themes and affective responses around patient experience and the resulted in a pool of potential items for further testing.

### Aim

The study aimed to explore patients’ experience of living with COPD and their perspective of their community healthcare for COPD, with the aim of extracting affective responses in order to develop potential items for a PREM for COPD.

While not the focus of this paper, the longer term aim is to continue refining the items through further testing to develop a short COPD PREM (10 items or less) to be used by the healthcare team in clinical practice to understand the positive and negative experience of living with COPD, cared for in a community setting, from the patient’s perspective.

#### Research questions

What are the main themes arising from analysis of patients’ accounts of their experiences of living with COPD, and of their community healthcare for COPD?

What are the affective responses contained within the themes, which describe the patients’ feelings and emotional experiences?

## Results

The sample reported comprised 64 patients with COPD, cared for primarily in a primary care setting. Forty of the participants were male (mean age = 71 years) and 24 were female (mean age = 73 years).

The themes and affective responses reported are not broken down by gender.

Five overarching themes were identified in the qualitative data analysis: (1) Journey to diagnosis; (2) Smoking; (3) Usual care; (4) My everyday life; and (5) Exacerbations. Within these themes a numbers of affective responses were contained. The themes with examples of narratives are given in Table 1, along with the affective responses within them.

**Table 1:** Themes and example quotes

Main themes	Subthemes	Selected quotations	
Journey to diagnosis		*‘In 2007 I started to feel very unwell and I wasn’t sure what it was. Went to my GP a number of times and was told it was a virus. I was told to get lots of rest and I would do that—and then I would go back to work feeling absolutely dreadful and a few days later I would be ill again. So this went on for about 3–4 months. Every time I went to the GP—‘Oh its just a virus’ I wasn’t given a chest x-ray absolutely nothing’*.	*‘ I’d never heard of COPD. I didn’t even know what it stood for […] it’s untreatable […] You have to learn to live with that’*.
Smoking		*‘Stopping smoking has been the best thing I ever did’*	*‘Dr. told me I had empysema and frightened the life out of me so I cut down on the cigarettes’*
Usual care	Communication	*‘I don’t think the staff went out of their way to tell me anything. I found it all out for myself, trial and error’*	*‘I know a lot about me lungs…I know what I’ve got. I know what’s wrong with me…I really have been educated’ (by COPD nurses & pulmonary rehabilitation)*
	Staff	*[COPD team have] ‘always been very helpful…there when I want them—that’s the main thing’*	*Can’t always get an appointment [with GP]…I don’t think he understands that when you are phoning (for a home visit or an appointment) it’s because you can’t breathe*
	Managing routine care	*‘I have got 2 inhalers but I don’t use regularly partly because I no longer get breathless. I no longer feel that I need. Although I have been told before that it doesn’t matter how I am feeling– I should be using them regardless there is something in me that doesn’t like having medication unless I really need it…I only tend to use them when my breathing is really bad and the only time when it is really bad is when I have a chest infection.’*	*‘I am slightly unsure (when to use inhaler)—at what point of incapacity do you feel in yourself…probably my fault for not asking’*
	Pulmonary rehabilitation	*‘Very good people there…gives you a reason to get up’*	*‘Some of it I thought was a total waste of space. Like standing against a wall and throwing a bottle [but] I gained something from it. Get companionship from other people…[but]to give me a 1 kg weight was an insult …[should be] tailored to the individual’*
My everyday life	Limitations	*‘Now I can’t even go up the stairs. It is shocking really. If I don’t have a couple of puffs. If I don’t take them and I’m out, and I have to move quick and it’s like drowning. You can’t get … it is the most awful sensation…..It is getting worse. Not stable it inches along. You know creeping over me. I could miss the puffers in the morning—1 day like in the morning but now I can’t. I have to take them’*	*‘I find sometimes if I go up an incline—I am not too bad going up stairs, we have go stairs—but if I go up an incline I tend to get out of breath. Just a ramp like the off ramp…..Sometimes it is very tiring. I came back from holiday last Tuesday night and on Friday I was cleaning out a bedroom and I was very tired out. Something that years ago would have 20 min—takes long time’*.
	Symptoms	*‘Sometimes I wake up in the night—I just can’t breathe. I have been told by the nurse that I should go to the hospital and have oxygen but I don’t though. I just—it passes and I manage to sleep……Yes—I have trouble breathing through my nose’*	*‘I know it is a silly thing—my husband was stripping paint because I wanted the paint stripped to varnish. Inhaling that gave me a cough of sorts and I tend to get a tickle and I did suffer a cough. I didn’t go the, I tend not to the Doctor—I though you can fight this. And that was bad it gave me a nasty cough. Fumes from cars give it to me and that cough persisted for a while. I was on a bus and if the doors are open the fumes… make me cough. Usually I get a tickle. I carry water’*.
	Labelling	*It’s embarrassing to tell others, I just say have another infection’*	*‘Family look at you and wonder what’s wrong with you. Haven’t told mum yet—like borderline cancer’*
	Life expectancy prognosis	*‘I don’t want to talk about the future, sounds so depressing—bad enough to be getting old.’*	*‘I Know people who have had empysema. They die. Gave me a bit of fright’*.
	Family job responsibilities	*‘My husband does a lot, he does housework; some cooking and I do try to walk’*.	*‘I live with my husband. He is 91 and also being looked by the practice*… *Also on care line. We have never need to use it—We have a panic button. They have keys to the house and they have our children’s numbers… And it is a great comfort that. Also with my husband—now he needs an actual carer’*
Exacerbation	Rescue pack	*‘NO—GP refused[to give rescue medication]… No—it is a joke—pretty diabolical. [GP is] insistent that come in. So if I get and infection on Friday night I then have to ring on Monday and get to see someone Wednesday. It is hopeless’*	*‘Nine times out of ten if anything is going to flare up it happens on a Friday night [so it]‘makes a big difference’*
	Access to staff	*Re. COPD team and GP ‘Very important knowing there is someone on the end of the line’*	*[COPD nurse is] ‘Kind and considerate and you don’t feel alone’*
	Emotional response to exacerbation	*‘Like having a plastic bag over your head…so frightening’*	*‘I worry about exacerbations and what to do’*

### Themes

#### Journey to diagnosis

This theme was about participants’ stories of being diagnosed with COPD which for some patients could be a slow journey of experiencing repeated respiratory infections before diagnosis or, for others, a sudden diagnosis often linked to an acute respiratory event.

This journey evoked mostly negative affective responses indicating ‘fear’, ‘frustration’ and ‘being surprised/shocked’.

#### Smoking

This theme centred on patients’ comments about their smoking behaviours including when they started smoking and when and why they continued or stopped smoking.

Affective responses included ‘self-motivation’ and the feeling ‘scared’.

#### Usual care

The focus of this theme was around patients’ experiences with community (primary) care, and comprised three sub-themes: ‘Communication’, ‘Staff’ and ‘Managing routine care’.

- ‘Communication’ was about obtaining information from health professionals and its relationship in communicating knowledge and understanding to patients about treatments and medications.
  This sub-theme often contained a number of negative affective responses but when patients felt they had sufficient information they felt ‘in control’ and ‘reassured’.
- ‘Staff’ was about responses participants experienced from various healthcare staff with whom they interact when receiving usual care for COPD.
  In terms of affective responses, the sub-theme contained positive affective responses, when patients were listened to and, therefore, felt ‘respected’ and felt ‘gratitude’ but also contained negative responses when there was perceived to be a lack of understanding in relation to communication leading to patients feeling ‘frustration’.
- ‘Managing routine care’ was about the patient experience of managing the treatments and medications for their routine COPD care, including their understanding of the rationale behind their treatments.

This could result in negative affective responses when patients felt ‘frustrated’ or ‘confused’ but more positive affective responses including being ‘self-motivated’ to manage their condition to prevent deterioration in their health.

Pulmonary rehabilitation was a particular element of routine care that evoked both negative affective responses of ‘frustration’ but also more positive affective responses of ‘enjoyment/ gratitude’ and ‘self-motivation’.

#### My everyday life

This large theme was about living with COPD, outside the medicalised aspect of the disease, and comprised various sub-themes.

- ‘Limitations’ was a sub-theme relating to living with COPD (activities of daily living). The negative affective responses connected to this theme were ‘frustration/annoyance’ e.g., ‘I get annoyed with myself, I can’t do what I want to’; but also the more positive affective response of ‘acceptance’ e.g., ‘Over the years I I’ve learnt to live with it’, and ‘gratitude’ and ‘hope’ around minimising those limitations.
- The sub-theme ‘Physical symptoms from COPD’, included accounts of the ‘ups and downs of living with COPD (Good days bad days).
  The negative affective response evoked by fluctuating physical symptoms were feelings of depression, e.g., ‘I get depressed because of going up and down, up and down [with COPD]’, and also ‘frustrated/annoyed’, ‘anxious’ and ‘worried’.
- The sub-theme ‘Diagnosis and labelling’ included accounts of embarrassment attached to having COPD, which were negative emotional responses e.g., ‘It’s embarrassing to tell others [about having COPD]’.
- The sub-theme of ‘Life expectancy/prognosis’ contained accounts of the knowledge that COPD is a deteriorating condition, which is highly likely to shorten the lifespan of sufferers. This sub-theme was associated with negative affective responses related to feeling depressed, e.g., ‘Talking about the future can be depressing’.
- The sub-theme ‘Responsibilities, family, job’ contained positive affective responses related to gratitude for help received from family and friends, e.g., ‘grateful for having a good wife…/help of partner’).

A strong affective response in both the theme ‘Usual Care’ and ‘Everyday life’ was the need to feel in control of COPD. This could carry either a positive, i.e., feeling in control, or negative, i.e., feeling out of control, valence and so was a bi-valent affective response.

#### Exacerbation

Persons with COPD may at some time experience an exacerbation in symptoms that may require additional treatment including rescue pack medications, containing antibiotics and steroids that the patient can start without consulting a doctor, and access to staff for help and management of their condition.

The broader theme of exacerbation itself was connected to strong negative affective responses, primarily fear and anxiety, e.g., ‘Like having a plastic bag over your head…so frightening’.

Sub-themes were;

- the use of rescue packs.
  The affective responses related to this sub-theme were mostly positive, with rescue packs providing a sense of ‘reassurance’ that treatment could be started quickly and when needed.
- Access to clinical staff and help during an exacerbation. This sub-theme contained accounts of which services were called upon, i.e., general practitioner (GP), paramedics, respiratory COPD team, and the perceived appropriateness or helpfulness of the response.

The affective responses to this theme varied and ranged from very positive feelings of gratitude and reassurance, e.g., [COPD nurse is] ‘kind and considerate and you don’t feel alone’, to very negative themes of anger, anxiety, fear and feeling depressed, e.g., ‘Who can I call?—I go to the doctor and he says you have to live with it’

### Affective responses

Twenty-one unique affective responses were identified from the data analysis and were categorised as (1) negative or (2) positive. The negative affective category included emotions such as ‘fear’, ‘frustration’ and ‘confusion’. While some patients indicated they had clinical depression, some used the word ‘depressed’ to indicate a negative feeling of ‘being down’ so we have used the term ‘feeling depressed’. Positive affective responses included ‘gratitude’, ‘hope’, ‘happy/enjoyment’. A few responses could be either negative or positive and so bivalent according to context. For example, ‘control’ could be positive ‘feeling in control’ or negative ‘feeling that it is out of control’.

‘Frustration’ was a prominent negative affective response, followed by the responses ‘annoyance/anger, ‘anxiety’ and ‘confusion’. ‘Gratitude’ was a prominent positive affective response found in several themes followed by ‘reassurance’.

Table 2 gives the themes and their associated affective responses, with some illustrative examples of patient quotations.

**Table 2:** Summary of affective responses and themes

Affective responses	Themes				
	Journey to diagnosis	Smoking	Usual care	My everyday life	Exacerbations
*Negative affective responses*					
Frustration	✓	—	✓	✓	—
Annoyance/Anger	—	—	✓	✓	✓
Anxiety	—	—	✓	✓	✓
Confusion	—	—	✓	—	✓
Fear/frightened	✓	—	—	—	✓
Feeling depressed	—	—	—	✓	✓
Scared	—	✓	—	—	✓
Embarrassment	—	—	—	✓	—
Surprise/shock	✓	—	—	—	—
Worry	—	—	—	✓	—
Feeling it is out of your control	—	—	✓	✓	—
Lack of respect	—	—	✓	✓	—
*Positive affective responses*					
Gratitude	—	—	✓	✓	✓
Reassurance	—	—	✓	—	✓
Acceptance	—	—	—	✓	—
Altruism	—	—	—	✓	—
Happy/enjoyment	—	—	✓	—	—
Hope	—	—	—	✓	—
Self-motivation	—	✓	✓	✓	—
Feeling in control	—	—	✓	✓	—
Feeling respected	—	—	✓	—	—

#### PREM items

Thirty-eight PREM-COPD items were generated from the patient experiences identified in this study. These were generated by examining the affective responses and formulating a statement that encompassed both the affective response and the thematic category in which the affective response arose. The statements were then worded in pairs to allow patients to score them from positive to the negative. For example: ‘I am confident that my doctor will listen to my point of view’ paired ‘I am concerned that my doctor won’t listen to my point of view’.

A summary of the affective responses and related themes is given in Table 3 and a full list of the 38 PREM items generated is given in Table 4.

**Table 3:** Themes affective responses and examples of patient narratives

Major theme	Explanation	Affective responses	Example a patient narrative
Journey to diagnosis	From the initial recognition by patients that they had symptoms to their diagnosis of COPD	• Fear • Frustration • Surprise/shock	*‘I kept seeing the GP (about symptoms) and I was getting frustrated… Eventually I saw a Dr. who said go for a Chest X-Ray* (which led to diagnosis)’. (Frustration)
Smoking	Some patients gave up smoking after they were diagnosed and others continued.	• Self-motivation • Scared	*‘I have stopped many times… the slightest bit of stress and I go back’.…* (Self-motivation)
Usual Care	Patient experiences with primary care		
		• Frustration • Annoyance • Confusion • Reassurance • Control • Anxiety	*‘Nobody tells you anything’*. (Frustration)
		• Respect • Gratitude	*‘’The GP says ‘You know better than we do’…* (Respect)
		• Enjoyment • Self-motivation	*‘The COPD clinic is very good… Have a good laugh’*. (Enjoyment)
My everyday life	The experience of living with COPD	• Frustration • Annoyance • Anxiety • Worry • Feeling Depressed • Embarrassment • Acceptance • Self-motivation • Gratitude • Hope • Altruism Respect • Control	*‘I can snap at people due to frustration (with physical limitations*)’. (Frustration/annoyance)
			*‘I’m not too bad… I’m not in bed every day so I’ve got a lot to be grateful for’*.
			(Gratitude)
Exacerbations	The experience of preventing and managing exacerbation of a patients’ COPD	• Fear • Scared • Anxiety • Annoyance • Anger • Reassurance • Confusion • Feeling Depressed	Trying to breathe in an exacerbation is ‘*Like having a plastic bag over your head… It’s so frightening’*. (Fear)

**Table 4:** Potential PREM items

MY HISTORY WITH COPD	
1 am not shocked by my COPD diagnosis	I am shocked by my COPD diagnosis
2 I have come to terms with my diagnosis of COPD	I have not come to terms with my diagnosis of COPD
3 I have given up smoking and I am confident that I will not start again	I have given up smoking but worry that that I might start again
4 I want to stop smoking and I believe that I can	I want to stop smoking and I believe I just can’t
5 It was a relief to have a diagnosis for my symptoms	Not having a diagnosis for my symptoms was frightening
USUAL CARE IN COPD	
6 I understand my diagnosis	I am confused about my diagnosis
7 I am confident that my GP will listen to my point of view	I am concerned that my GP would not listen to my point of view
8 I am very pleased with healthcare workers who look after my COPD that I see on a regular basis	I am not at all pleased with healthcare workers who look after my COPD that I see on a regular basis
9 I am happy with the length of time that it takes to get an appointment with my GP when I need to	I am angry about the length of time that it takes to get an appointment with my GP
10 I really enjoyed pulmonary rehabilitation	I didn’t enjoy pulmonary rehabilitation
11 I found pulmonary rehabilitation useful	I didn’t find pulmonary rehabilitation useful
12 I understand my condition and this helps me to manage my fear	My lack of understanding about my condition makes me frightened
13 The information I have been given from the healthcare workers about my COPD is consistent	I have been given conflicting information from the healthcare workers about my COPD
14 I have enough information about my condition	I am frustrated by my lack of information about my condition
15 I understand about my COPD tablets	I am confused about my COPD tablets
16 I understand how to use my COPD inhalers	I am confused about how to use my COPD inhalers
17 I understand about how my COPD treatments work	I am confused about how my COPD treatments work
18 I don’t find going to a hospital outpatient clinic frustrating	Going to a hospital outpatient clinic is frustrating
19 I know how to use my inhaler properly	I am confused how to use my inhaler properly
MY EVERYDAY LIFE WITH COPD	
20 I have accepted the limitations to my lifestyle caused by COPD	I am frustrated and unhappy with the limitations to my lifestyle caused by COPD
21 I feel that I have good support from others like my family, friends, neighbours or carers	I feel that I have do not have any support from others like family, friends, neighbours or carers
22 Overall I am satisfied with my life	Overall I am dissatisfied with my life
23 I am not depressed	I am feeling depressed
24 Overall I am satisfied with the care given to me	Overall I am dissatisfied with the care given to me
25 I am not embarrassed to tell others about my condition	I am embarrassed to tell others about my condition
26 I feel that I am in control of my condition	I feel that I do not have any control over my condition
27 I am motivated to keep going and not give up	I am not motivated and I feel like giving up
28 I am happy to talk about the future	Talking about the future makes me depressed
29 I am not concerned about the future	I am concerned about the future
30 I am not concerned about the season	I worry about the season and my COPD
31 I keep going and try to enjoy my life	I feel like giving up and I do not enjoy my life
COPD EXACERBATION (FLARE UP)	
32 I am confident in a ‘flare up’ I have quick access to treatment, e.g., a rescue pack or access to my GP	I am worried that in a ‘flare up’ I do not have quick access to treatment, e.g., a rescue pack or access to my GP
33 I do not feel anxious about my current health	I feel anxious about my current health
34 I am not worried about the care I will get from health professionals when I have a ‘flare up’	I worry about the care I will get from health professionals when I have a ‘flare up’
35 I am not scared of getting a cold or an infection	I am scared of getting a cold or infection
36 I am not frightened of being breathless when I have a ‘flare up’	I am frightened of being breathless when I have a ‘flare up’
37 I am not frightened to go to sleep when I am having a ‘flare up’ of my COPD	I am frightened to go to sleep when I am having a ‘flare up’ of my COPD
38 I try not panic when I have a ‘flare up’ as it will make my breathlessness worse	I panic when I am having a ‘flare up’ and this makes my breathlessness

## Discussion

A patient-centric approach to healthcare requires the patient voice to be present when we assess a patient’s experience with their health and interaction with healthcare organisations. Hodson and Andrew^2^ state: ‘To borrow from qualitative research, there are multiple views of reality: patients may prioritise care differently, view events differently and have different opinions about what is important to them when interacting with nurses, other healthcare workers and the healthcare system.’

Patient satisfaction scales and quality of life measures have traditionally ignored emotions associated with patient experiences when living with long-term conditions. We need new approaches to capturing patient experiences including how they view and describe an experience from their viewpoint using those emotions and words patients use when describing their experience.^1, 2^

The fear or emotive response COPD patients have to the experiences of shortness of breath, which is exacerbated in an acute episode of COPD, is well recognised by practitioners. Our study indicates the experience of living with long-term condition such as COPD evokes a wide range of emotions in patients. These emotions which we term ‘affective responses’ encompass both negative and positive responses.

In our approach to the development of our preliminary items for the PREM, we sought to capture the emotions and words used by patients in the items. We used a two-step analysis to enable us to categorise the experiences of patients, firstly using themes and sub-themes, and secondly to identify underlying participant emotions from the tone, accessible directly from the audio-files, and expressions used in relaying their experience living with COPD, and interacting with the healthcare system. We feel that our method of analysis, working directly from audio-recordings, enhanced our appreciation of the affect being expressed in the interviews.

### Limitations and strengths

In contrast to quantitative research approaches, qualitative research does not seek generalise results to the population and sample numbers are frequently small. However, the sample numbers were a strength in this qualitative study with an adequate number of patients recruited and interviewed which allowed us to approach data saturation. We had been advised by the Picker Institute, who has developed the UK national patient survey tool and other similar devices, that using patient life-story (discovery interview) techniques as used here, a sample size of <20 is usually sufficient to produce a data saturation point. We anticipated about a 50% drop out rate for COPD subjects who volunteered to participate in advance due to ill health and or conflicting engagements so increased the number of participants we planned to invite to participate. This resulted in a sample size of 64 participants, which exceeded our requirements for data saturation.

The use of pre-registration nursing students as data collectors was an effective way of conducting a large number of interviews and, while they had limited knowledge of COPD, they were adept at listening to a patient’s story, due to their training in the discovery interview technique. A limitation, however, of using non-expert interviewers was their lack of experience in asking follow-up questions about certain aspects of care raised by patients. The use of multiple interviewers to gather data might also present a limitation to the quality and consistency of the data collected. However the ‘discovery interview’ technique used requires very little input from the interviewers, after the initial stimulus question. While variation in responses due to inter-personal factors cannot be excluded, we feel the use of multiple interviewers, for eliciting patient experiences, using discovery process interview techniques, is unlikely to have introduced significant variations in the data.

The interviews reveal rich data about patients’ experiences of living with COPD indicating the effectiveness of the data collection methods. While qualitative data collection has limitations, we adhered to the rigour expected in qualitative research to maximise the quality of the data collection and analysis.

Patient recruitment was clearly focused on targeting patients with COPD who self-selected to join the study. Questions about diagnosis and confirmation that patients had COPD were asked at interview, and while we are confident that patients did have COPD, we acknowledge the lack of formal confirmation as a limitation in this study. Similarly, we did not conduct spirometry measurements, which would confirm the presence and severity of a patient’s COPD.

Interviews were conducted in English only and, therefore, results may not be applicable linguistically diverse patients. There is some evidence that the self-reported experiences of patients in the UK NHS (National Health Service) differ by ethnic groups,^14^ and so the lack of linguistic diversity in our sample is likely to be an important limitation.

This research involved community-based patients, who are likely to have less severe COPD than hospital-based patients. This PREM is, therefore, intended to be used in a community-based setting.

### Future developments

The second stage of the development of the PREM for COPD is underway with the aim of reducing the 38 items from the first phase to a scale of <10 items. COPD patients in this phase of the study will have spirometry testing to confirm the diagnosis of COPD. The final version of the measure will enable us to measure their experience of living with COPD and healthcare interactions in association with other measures such as PROMS.

A systematic tool for collecting patient experience, such a COPD PREM, has potential to improve the quality of the service provided for COPD patients in a community setting. On an individual basis the responses to a COPD PREM may allow a dialogue to open up between the patient and the healthcare provider, which can address the emotional impacts of living with COPD, and potentially alleviate some of the negative experiences such as frustration about accessing GPs in a timely manner. It can also provide a stimulus to discuss patients’ feelings about stopping smoking, or to provide more factual information, if that is desired. Where a completed PREM indicates ongoing levels of fear and anxiety, signposting to specialist services may be indicated. On a service provision level, the use of a COPD-specific PREM may illuminate gaps in service provision, which commissioners can address, and provide evidence for the continuance of good quality services, which patients’ indicate offer reassurance and provide confidence in their care.

In this context, the PREM may enable us to understand the patient experience at the time of measure and to better plan healthcare interactions accordingly.

## Conclusions

Our work towards the development of a PREM for COPD has enabled us to move the measurement of the patient experience from the traditional medical model with a focus on healthcare expectations to those focusing on the patient, capturing their words and experiences. The need for such a measure derives from a bio-psycho-social understanding of patients receiving healthcare, which acknowledges that a narrow focus on measurable outcomes or generic satisfaction does not fully capture those aspects of care that are important to patients, and so provide good experience of healthcare services.

## Methods

Mixed methodological approaches are increasingly being used to research healthcare issues due to their ability to capture some of the complexity of these issues.^15^ A sequential exploratory research design is one that is particularly employed in the development and testing of a research instrument. This project used a mixed method sequential exploratory design to develop a PREM for patients with COPD. The first stage of PREM development, reported in this paper, involved the conduct of qualitative interviews with patients to inform the construction of a bank of preliminary items for the tool.

### Setting and ethics

The study was conducted in East London and the City, Outer North East London and Essex regions of the UK.

Methods were performed in accordance with relevant regulations and guidelines.

National Health Service Research Ethics Committee approval was obtained for the study (Stanmore, London: Ref. no.11/LO/0714). Participants gave fully informed, written consent to participate.

### Sample

Sixty-four patients with a diagnosis of COPD, were interviewed for this study. The sample comprised 40 (62.5%) males with a mean age of 71 years and 24 (37.5%) females with a mean age of 73 years.

The sample was recruited from GP practices, Breathe Easy groups and community respiratory teams and thus comprised a sample of patients being cared for primarily in the community, as opposed to the hospital setting. Breathe Easy support groups are found throughout the UK, and are aligned with The British Lung Foundation, a UK charity. Community care of COPD patients is free to NHS patients, and comprises care provided by GP practices and teams of respiratory nurses.

General practice and community respiratory teams sent out invitations to participate, to patients registered on their COPD lists that they deem to be suitable. Those invited to take part were registered as having COPD on the GP register. Patients were excluded if the GP or clinical team considered the patient to be: vulnerable and/or unable to provide informed consent for participation; if the patient had another terminal disease; or if the patient was unable to communicate in spoken English.

The interviewees comprise a self-selected sample of those invited to attend. Confirmation that patients had COPD was obtained verbally at interview.

The audio-recorded interviews were conducted in community locations and patient homes within the selected regions. Patients were interviewed alone or with family members present according to patient choice. The interviews were audio-taped and lasted approximately 45 min. Interviews consisted of two elements. The first element, lasting approximately 30 min, used discovery interview (patient story) techniques^16^ and were loosely structured around healthcare interactions as a result of their COPD diagnosis. These types of interviews are largely unstructured and aim to allow telling of the patient’s story about a particular experience or event, with minimal prompts used to ensure the focus of the interview remains the experience of the condition, in this case, COPD. In this project, the patient was asked to recount experiences of healthcare interactions related to COPD and to emphasise the issues that were fundamental to their experience of COPD care. In the latter 15 min the patients were asked about the importance of a number of experience items, derived from a literature search of PREMS content items, for example pulmonary rehabilitation, medication and end-of-life care. This stimulated further accounts of patient experience in some participants.

#### Interviewers

Pre-registration graduate nursing students from a local university were given an overview and educational session on COPD and were trained in interview technique by the Picker Europe Institute [http://www.picker.org]. The students were studying research as part of their nursing curriculum and this project gave them the opportunity to participate in conducting an aspect of research. Approximately, ten pairs of students conducted the interviews in this sample.

### Data analysis

Data analysis was undertaken by two researchers and steps were undertaken to establish rigour in the data analysis process. After an independent analysis of three separate interviews by each researcher, codes were agreed, and the codes applied to the remainder of the interviews were compared and discussed at intervals, in order to ensure consistency.

The analysis of the data was conducted using a two-step approach—Step 1—a preliminary thematic analysis, concerning the content of the interviews, and Step 2—a subsequent analysis of the affective experiences, contained within the themes.

In order to preserve the authenticity of patients’ affective responses in the interview data, analysis of the interview data was undertaken directly from the interview audio-files.^17^

The preliminary thematic analysis (Step 1) was guided by Coffey and Atkinson^18^ and Mathieson and Barrie.^19^ This phase of data analysis was primarily concerned with the *content* discussed by patients in their interviews.

**Fig. 1:**
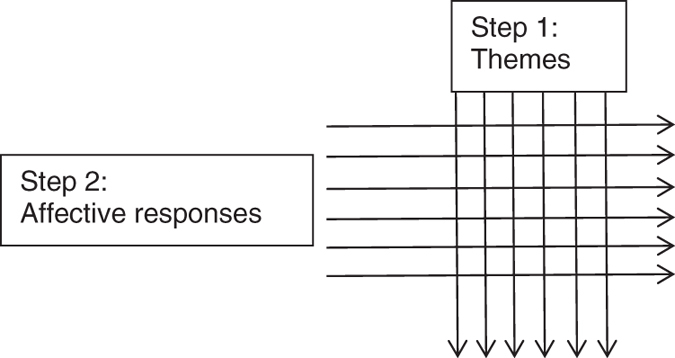
*Schematic view* of data analysis

In Step 2 of this analysis, the already coded data were analysed and coded again, structured by the thematic categories, and re-coded to extract the *affective* responses related to the thematic content in patient interviews. The resulting analysis, therefore, has two layers. The first layer relates to content and the second layer relates to the affective responses attached to the experiences. The affective responses can be viewed as cutting across the themes, and providing the emotional aspect of the experiences reported (Fig. 1).

**Fig. 2:**
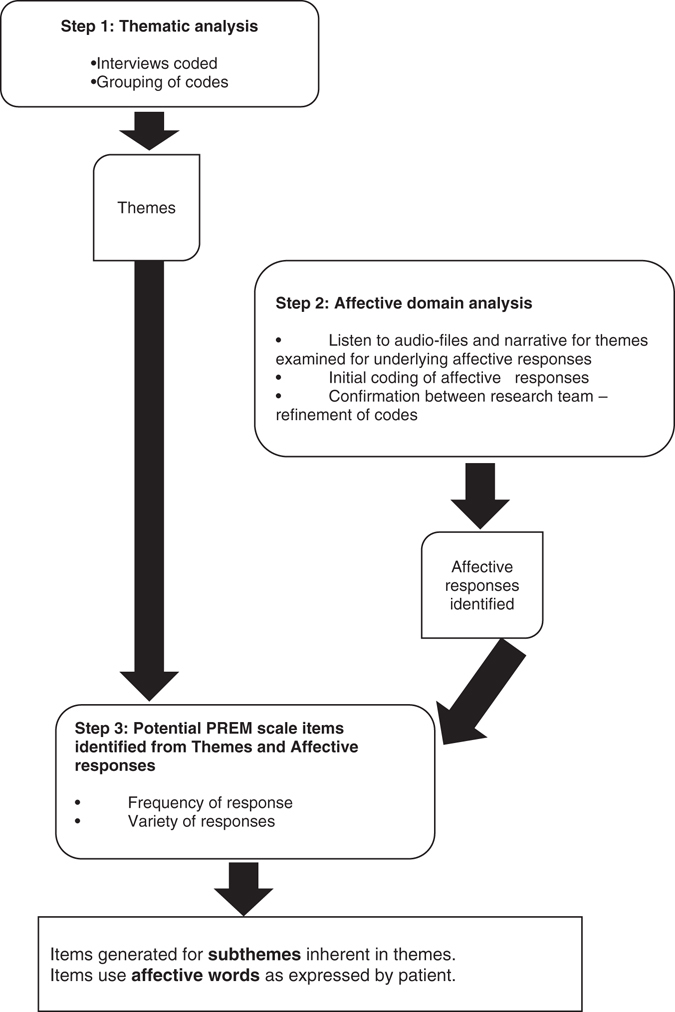
*Steps* in data analysis

The sequence of data analysis is presented in Fig. 2.

### Data availability

We have not obtained consent for the data under-pinning this research to be made freely available on request, due to its qualitative and highly personal nature.
